# Information Retention and Feature Screening Synergistic Network for Aviation Ground Safety and Protective Devices

**DOI:** 10.3390/jimaging12080386

**Published:** 2026-08-17

**Authors:** Enming Wu, Mingxuan Wang, Runxia Guo, Jiusheng Chen, Jiaren Li, Fuyu Sun, Liyuan Ye

**Affiliations:** 1 Engineering and Technical Training Center, Civil Aviation University of China, Tianjin 300300, China; 2 School of Electronic Information and Automation, Civil Aviation University of China, Tianjin 300300, China; 3 Department of Maintenance & Engineering, Qingdao Airlines Co., Ltd., Qingdao 266200, China; 4 School of Aeronautical Engineering, Civil Aviation University of China, Tianjin 300300, China

**Keywords:** object detection, aviation ground safety and protective devices, information bottleneck theory, dual-branch network

## Abstract

Aviation ground safety and protective devices are critical for flight safety; however, their unintentional retention on aircraft after maintenance remains a persistent risk. Existing deep learning-based approaches for aviation safety have predominantly followed a reactive paradigm, detecting FOD on runways or inspecting the aircraft for inadvertently retained tools post-maintenance. In contrast, this paper advocates a proactive philosophy: using a neural network to recognize and inventory all ground safety and protective devices immediately after maintenance closure, thereby preventing retention incidents at their source. However, realizing this proactive verification is technically challenging—object detection for these devices often suffers from loss of fine-grained detail due to downsampling and inherently sparse semantic information of the targets. To this end, we propose an Information Retention and Feature Screening Synergistic Network (RS-Net) grounded in information bottleneck theory. The network comprises a main branch that enhances discriminative features through attention-guided screening, and an auxiliary branch, used only during training, that preserves fine-grained spatial details via information-retentive convolutions. A Dual-State Region Refinement Module (DRM) provides configurable support for both branches, decoupling the conflicting objectives of background compression and detail preservation. Experiments on a self-constructed dataset collected from real airline maintenance operations demonstrate that RS-Net substantially outperforms the strong YOLOv9 baseline, achieving gains of 4.531% in F1-score, 2.533% in mAP_0.5_, and 1.429% in mAP_0.5:0.95_. Cross-dataset experiments further validate its strong generalization capability.

## 1. Introduction

The management and inventory of aviation ground safety and protective devices are critical for ensuring aviation safety. In the context of aircraft maintenance, the unintentional retention of protective devices, such as pipe sleeves and landing gear pins, significantly compromises the measurement accuracy of airborne instruments and the reliability of landing gear retraction systems. If such items are overlooked on the airframe or tarmac, they pose a critical threat to flight safety, often necessitating emergency procedures such as air turn-backs. Traditional methods that rely on human memory and handwritten logs are regulated by procedures, but they suffer from inherent efficiency and reliability issues, entailing high labor costs for airline operations. Consequently, exploring and applying intelligent, automated tool detection technologies has become an inevitable trend in enhancing the safety management of aviation maintenance, with the aim of building a more robust defense system.

Recent years have witnessed substantial advances in object detection, with models becoming increasingly proficient at achieving high performance on standard datasets such as Microsoft Common Objects in Context (MS-COCO) and PASCAL Visual Object Classes (PASCAL VOC) [1,2]. However, these mainstream datasets do not include the specific category of aviation ground safety and protective devices, which makes it difficult to replicate existing models directly in this domain. This limitation stems from two main factors. First, datasets specifically constructed for aviation ground safety and protective devices detection are extremely rare. Second, the task itself presents unique challenges: aviation ground safety and protective devices are typically small, which makes features prone to being lost during model downsampling. Additionally, their low correlation with the image background results in a lack of effective contextual cues.

To address these challenges, this study creates a realistic and efficient aviation ground safety and protective devices dataset based on the daily maintenance operations of an airline. This dataset closely aligns with practical application scenarios and provides crucial data support for subsequent research. Building upon this foundation and inspired by the information bottleneck theory, which highlights the trade-off between compressing background noise and preserving target-critical information, we propose an Information Retention and Feature Screening Synergistic Network (RS-Net) based on YOLOv9, incorporating a Dual-State Region Refinement Module (DRM) to enhance the detection performance of aviation ground safety and protective devices.

**Figure 1:**
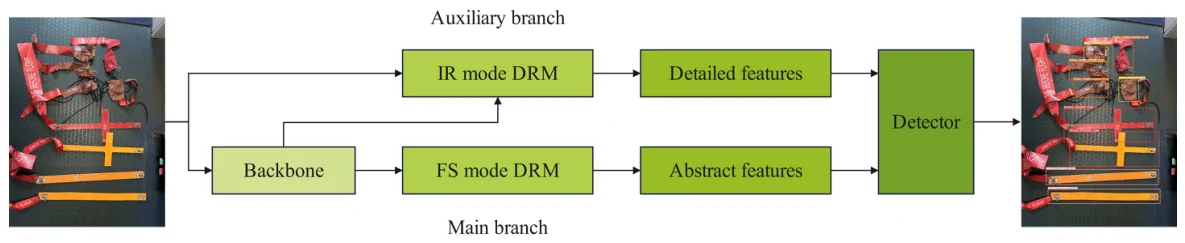
The RSNet consists of two branches and adopts two types of DRM modes. The auxiliary branch uses the IR mode of DRM to extract detailed features, while the main branch uses the FS mode of DRM to extract abstract features. The detection head receives the feature maps from both branches and supplements the details in the feature map of the main branch, guiding the main branch to more accurately select features.

As shown in Figure 1, The RS-Net comprises two branches: a main branch dedicated to detection tasks, and an auxiliary branch that is designated for use only during training. The main branch uses pooling layers that are good at screening features and multiple attention mechanisms that focus on and enhance feature representations in critical regions. The auxiliary branch employs convolutional layers optimized for information fidelity to preserve the complete gradient information flow. During training, this auxiliary branch acts as an implicit knowledge distillation mechanism, guiding the main branch to select the correct and complete features of the detection targets. This enables the model to support and enhance the main branch during training, effectively mitigating information loss in small objects caused by downsampling.

The DRM is the core feature extraction module of the RS-Net and has dual operational modes. When embedded in the main branch, it activates multiple attention mechanisms to strengthen critical regions. When embedded in the auxiliary branch, it disables any operations that could compromise information integrity, ensuring reliable gradient feedback. This collaborative, dual-branch design of the RS-Net effectively addresses the unique challenges of aviation ground safety and protective devices detection, enhancing the model’s detection performance comprehensively.

The main contributions of this study can be summarised as follows:

- First, grounded in information bottleneck theory, we propose RS-Net to explicitly decouple the inherent conflict between background compression and detail preservation: the main branch suppresses task-irrelevant interference via attention-guided screening, while the auxiliary branch preserves fine-grained spatial gradients via information-retentive convolutions, thereby approximating the optimal information-bottleneck trade-off.
- Second, we design a Dual-State Region Refinement Module (DRM) with switchable Feature Screening (FS) and Information Retention (IR) modes tailored for the dual-branch framework: FS mode employs multi-scale attention to enhance discriminative features, while IR mode disables attention to retain low-level spatial details, directly compensating for the detail loss caused by downsampling in small-target detection.
- Third, extensive experiments on a real-world airline maintenance dataset demonstrate that our RS-Net outperforms the strong YOLOv9 baseline by absolute margins of 4.531% in F1-score, 2.533% in mAP_0.5_, and 1.429% in mAP_0.5:0.95_, with cross-dataset validation further confirming its robust generalization capability.

## 2. Related Works

Thanks to the advancements in deep neural networks, object detection technology has been widely applied in numerous fields. Following the widely adopted taxonomy in recent surveys [3], current object detection algorithms are mainly divided into three categories: two-stage detectors such as Faster R-CNN and Mask R-CNN [4,5], one-stage detectors such as the YOLO series [6,7,8,9,10] and SSD [11], and Transformer-based detectors such as DETR [12] and RT-DETR [13]. Two-stage algorithms typically first extract candidate regions before performing classification and regression, aiming for higher detection accuracy; whereas one-stage algorithms directly complete localization and classification in a single forward pass, emphasizing real-time performance and detection speed. Transformer-based detectors adopt an end-to-end paradigm that eliminates the need for hand-crafted components like non-maximum suppression (NMS) and offers greater potential for architectural scalability and cross-task generalization. Some studies have optimized the loss functions of neural networks to address the specific challenges of object detection. For example, Yu et al. proposed the first object detection network trained directly using IoU as the loss function [14]. Zheng et al. introduced a penalty term based on the distance between the centers of predicted and ground truth boxes to the IoU loss, achieving faster convergence and better localization accuracy [15]. Rezatofighi et al. proposed a generalized IoU metric and loss function that addresses the vanishing gradient issue in IoU loss when predicted and ground truth boxes do not overlap by incorporating the smallest enclosing region [16]. Ma et al. proposed an IoU loss that minimizes point-to-point distances to more comprehensively consider the geometric relationships of bounding boxes, enabling efficient and accurate regression [17]. Tong et al. proposed a bounding box regression loss with a dynamic focusing mechanism, which dynamically adjusts gradient gains by evaluating anchor quality to mitigate the negative impact of low-quality samples [18]. Wang et al. proposed a prediction probability loss function (PP-Loss) based on object size to optimize the classification loss in multi-scale object detection networks, thereby improving small object detection performance [19]. Shi et al. proposed a joint optimization loss function (JOL) that enhances the accuracy and balance of small object detection in remote sensing images by dynamically weighting classification and regression losses [20].

In the field of aviation defect detection, many studies have proposed improvements to address challenges such as small objects, complex backgrounds, and data scarcity [21]. For instance, Huang et al. proposed ASD-YOLO, which enhances the extraction capability for irregular corrosion features on aircraft skins through deformable convolution and a global attention mechanism [22]. Qu et al. integrated the Biformer attention mechanism and normalized Wasserstein distance loss into YOLOv5, significantly improving the detection accuracy of small corrosion defects [23]. Wang et al. introduced lightweight improvements to YOLOv8n by incorporating the Shuffle Attention++ module and a bidirectional feature pyramid, achieving higher recall and average precision while maintaining a high frame rate [24]. Li et al. integrated a deformable convolutional network into YOLOv5s to enhance the adaptability of feature maps to shape variations, thereby improving the accuracy of engine damage detection [25]. Chen et al. improved YOLOv8n using knowledge distillation to compress model size and computational complexity while preserving detection accuracy [26]. Ding et al. enhanced Mask Scoring R-CNN with an attention mechanism to improve pixel-level segmentation capability for corrosion edges [27]. Fu et al. achieved high recognition accuracy with limited samples through transfer learning combined with pre-trained CNN models [28]. Zhang et al. introduced a multi-scale fusion attention mechanism into DETR, combined with R-focal loss to alleviate sample imbalance issues, achieving an AP of 92.8% in aircraft engine blade defect detection [29]. For the aircraft surface defect detection task, Zhang et al. introduced coordinate and channel attention mechanisms along with an adaptive path aggregation network based on YOLOv7. By coordinating multi-scale feature fusion and optimizing the loss function, they improved the detection accuracy of multi-scale defects under complex backgrounds [30]. Upadhyay et al. proposed a deep learning-based framework for aircraft engine defect detection, achieving high-precision automatic endoscopic inspection through a customized U-Net and a hybrid motion deblurring method [31]. Zhang et al. presented an improved YOLOv5-OBB model based on an attention mechanism. By incorporating channel-spatial attention modules and a feature pyramid non-local module, they enhanced the detection accuracy of directional surface defects during aircraft skin coating processes [32]. Wen et al. designed a Multi-Domain Feature Alignment Network (MDFANet), integrating multi-scale feature fusion and an image-level domain adaptation module within the YOLOv7 framework, which effectively improved the detection capability for minute defects on aircraft engine turbine blades under small-sample conditions [33].

Beyond surface defect inspection, object detection techniques have also been applied to aviation ground safety scenarios. In runway Foreign Object Debris (FOD) detection, which targets unintended objects including tools and aircraft components that pose severe hazards during takeoff and landing, several YOLO-based detectors have been proposed. Zhang et al. developed SPLP-YOLO, which integrates spatial-to-depth convolution and large-kernel attention to preserve small-target details in all-weather conditions [34]. Wang et al. proposed YOLO-URD, a lightweight UAV-based model that achieves high precision with minimal parameters for runway FOD detection [35]. Liu et al. introduced VA-YOLO, which incorporates vision transformer and attention mechanisms to address misdetection and omission issues in complex airport environments [36]. These studies collectively demonstrate the effectiveness of CNN-based methods for object detection in airport settings. Beyond FOD detection, Chen et al. specifically addressed the detection of aircraft landing gear safety pins using an improved YOLOv5s model, achieving notable improvements in both accuracy and efficiency [37]. This work confirms that ground safety and protection devices are viable targets for deep learning-based detection.

Nevertheless, the above approaches predominantly follow a reactive paradigm: FOD detection identifies foreign objects already present on runways, while safety pin detection verifies the removal of devices after maintenance work is completed. Both operate at the end of the risk chain, detecting hazards that have already been introduced. In contrast, the present work adopts a proactive philosophy—shifting the risk control point forward to the moment when maintenance concludes. Rather than waiting for a pre-flight inspection to discover a forgotten device, our approach directly verifies, at the point of maintenance closure, that all ground safety and protection devices—including static pressure port covers, total temperature sensor covers, landing gear ground lock sleeves, and pitot tube covers—have been properly removed and retrieved. This paradigm shift from “detect after the fact” to “prevent at the source” fundamentally distinguishes our approach from existing FOD and tool management studies. However, comprehensive research targeting this full range of aviation ground safety and protection devices remains scarce, which constitutes the research gap that the present study seeks to fill.

Regarding challenges in the aviation maintenance inspection field, research from other domains also offers valuable insights. For instance, to address feature loss in small object detection, Qu et al. designed a Feature Fusion and Distribution Module (FFDN) based on YOLOXs, which effectively enhances feature representation and detection performance for small objects through global feature fusion and redistribution mechanisms [38]. Zhou et al. proposed a dual-path lightweight attention network that extracts high-level semantic and low-level detailed features in parallel, combined with lightweight self-correlation and cross-correlation modules. This approach significantly improves real-time object detection accuracy while maintaining high efficiency [39]. Xu et al. constructed the HCF-Net (Hybrid Convolutional Fusion Network) using PPA, DASI, and MDCR modules, effectively boosting the model’s capability in detecting small objects [40].

General datasets are unsuitable for model training in specialized domains, making the construction of high-quality, domain-specific datasets an essential step for object detection tasks in such fields. Regarding dataset development, researchers have successively proposed high-quality, diverse specialized datasets across multiple domains. For example, Kharismawati et al. proposed the MSDD dataset, focusing on corn seedling detection and covering different growth stages, camera angles, soil colors, and planting densities, providing an important benchmark for agricultural visual inspection [41]. Chen et al. constructed the Aircraft_Fuselage_DET2023 dataset for semi-supervised detection of aircraft fuselage defects (such as scratches, rust, and rivet damage) and alleviated the issue of insufficient labeled data through a category-adaptive pseudo-label assignment strategy [42]. Vashist et al. proposed the FOD-S2R dataset, focusing on foreign object detection inside aircraft fuel tanks. It includes both real collected images and synthetic images generated using the Unreal Engine, supporting Sim2Real transfer learning research and improving detection generalization in closed, complex environments [43].

## 3. Methodology

**Figure 2:**
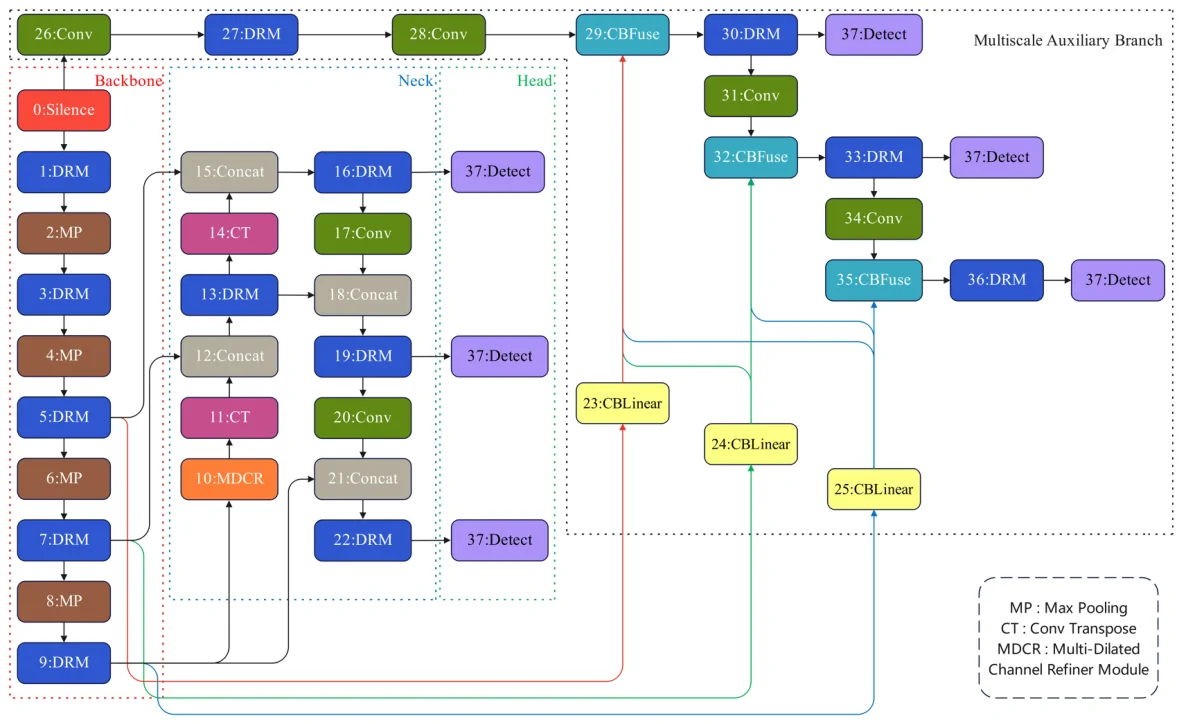
The RS-Net network structure. The network consists of two branches. The main branch extracts abstract features through DRM and pooling layers in the feature selection mode, while the auxiliary branch extracts detailed features through DRM and convolutional layers in the information retention mode. Both the main and auxiliary branches will obtain feature pyramids of the same scale to be input to the detection head.

This section details the architecture of RS-Net. As illustrated in Figure 2, RS-Net is built upon a path aggregation network, augmented with an auxiliary branch. The main and auxiliary branches are trained concurrently with iterative parameter updates. During inference, only the main branch is retained to ensure efficient processing. A key component is the novel Dual-state Region Refinement Module (DRM), which is designed to enhance feature extraction for small objects. The workings of the dual-branch architecture and the DRM are elaborated in the following three subsections, along with the complete specification of RS-Net.

### 3.1. Information Bottleneck Theory in Object Detection

According to Information Bottleneck theory, the deep learning process can be formulated as optimizing an intermediate representation T of the input data X [44]. This representation aims to maximize compression of task-irrelevant information in X while preserving maximal mutual information with respect to the output label Y, as formalized in Equation (1) [45]:

(1) L[p(t|x)]=[I(X;T)−βI(T;Y)],

where L[·] denotes the Lagrangian function to be minimized, β denotes the Lagrangian multiplier, and p(·|·) denotes the conditional probability distribution. β denotes the Lagrangian multiplier that controls this trade-off: as β approaches 0, the model prioritizes retention of information pertinent to Y; conversely, as β approaches infinity, emphasis shifts toward compressing redundant information in X.

The information bottleneck theory elucidates an inherent trade-off in object detection: the tension between preserving task-relevant information and compressing irrelevant details. Mathematically, as shown in Equations (2) and (3) [7]:

(2) I(X;f(X))=I(X;X)=H(X),

(3) I(X;g(X))<I(X;X)=H(X),

where H(·) denotes the information entropy. An invertible mapping f(·) ensures that the output f(x) retains complete information from the input x, whereas non-invertible transformations g(·) inevitably incur information loss. While architectures such as RevNet and NICE achieve exact reversibility at the module level [46,47], network-wide invertibility imposes stringent constraints on memory footprint and parameter efficiency. Consequently, most CNN structures, being non-invertible in the strict sense, suffer from progressive information degradation as depth increases. Although this process effectively suppresses irrelevant information, it concurrently attenuates a fraction of critical information required for object detection. Thus, the Information Bottleneck framework conceptualizes this dilemma as an information-theoretic optimization problem balancing preservation against compression.

**Figure 3:**
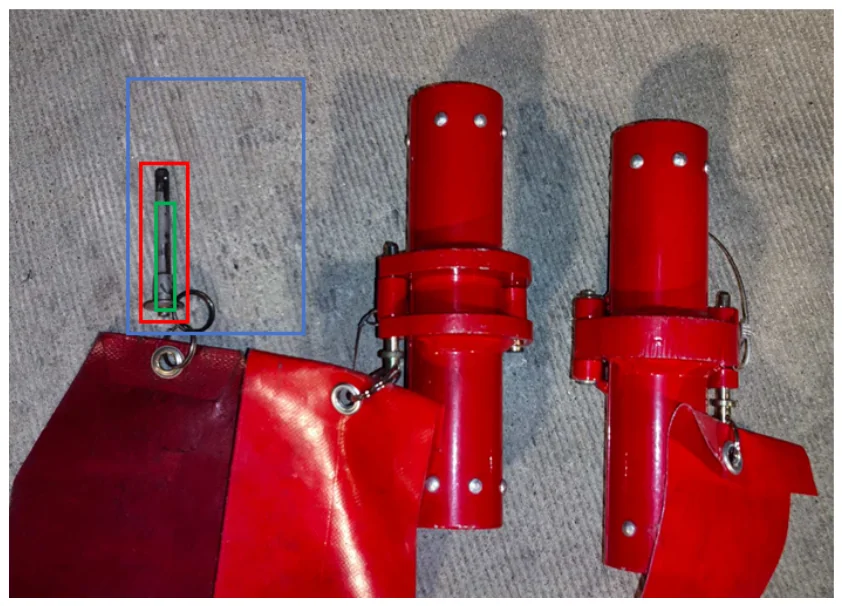
Visualization of information bottleneck theory. The red bounding box denotes the ground truth. The optimal intermediate representation *T* should preserve high mutual information with the target I(T;Y) while compressing irrelevant information from the input I(X;T). The blue and green boxes illustrate two suboptimal cases that fail to balance this trade-off: the blue box is I(T;Y)≈I(X;Y), but I(X;T) still remains at a relatively high level, indicating that the background compression is insufficient; conversely, the green box is different. I(X;T)≈0 indicates that the background has been effectively filtered out, but I(T;Y)<I(X;Y), meaning that some of the target information has been lost.

As illustrated in Figure 3, obtaining the optimal representation T necessitates simultaneously minimizing I(X; T) and maximizing I(T; Y), which constitutes a formidable optimization challenge. Shallow architectures exhibit limited capacity for feature abstraction, whereas deeper networks are prone to excessive information loss; notably, specific module designs differentially affect information propagation. Conventional approaches typically excel at either reducing I(X; T) or maintaining I(T; Y), but rarely both. To address this limitation, we propose a dual-branch architecture to approximate the optimal representation: an auxiliary branch dedicated to preserving fine-grained spatial details, and a main branch performing deep abstraction to accentuate salient features. Both branches employ independent scoring mechanisms to generate dynamic positive sample assignments, thereby establishing implicit knowledge distillation during gradient backpropagation. This configuration enables the main detection head to focus on abstract feature extraction via its dedicated loss function, while the auxiliary branch assumes responsibility for detail preservation. Through this cooperative mechanism, the network progressively converges toward the optimal representation T.

### 3.2. Dual-State Region Refinement Module (DRM)

The information bottleneck theory suggests that for object detection, valuable information must be preserved while irrelevant data is compressed. However, it is highly challenging to train a single module to simultaneously maximize I(T;Y) and minimize I(X;T). This dilemma is particularly acute in our task: the fine-grained details of aviation ground safety and protective devices prone to being lost during downsampling, while their low correlation with the image background results in a lack of effective contextual cues. Therefore, we design a dual-mode module (DRM) that adaptively operates in two distinct states. The Information Retention (IR) mode preserves spatial details to address the first challenge, while the Feature Screening (FS) mode employs multiple attention mechanisms to enhance target features and suppress irrelevant background, addressing the second. By decoupling these two objectives into separate modes, the DRM provides flexible component-level support for our dual-branch framework. The DRM operates in two states: Information Retention (IR) and Feature Screening (FS). In IR mode, the DRM uses a simpler architecture that primarily relies on convolutional layers to process feature maps. In FS mode, the architecture becomes more complex, incorporating numerous attention layers alongside convolutional layers to enhance critical regions.

**Figure 4:**
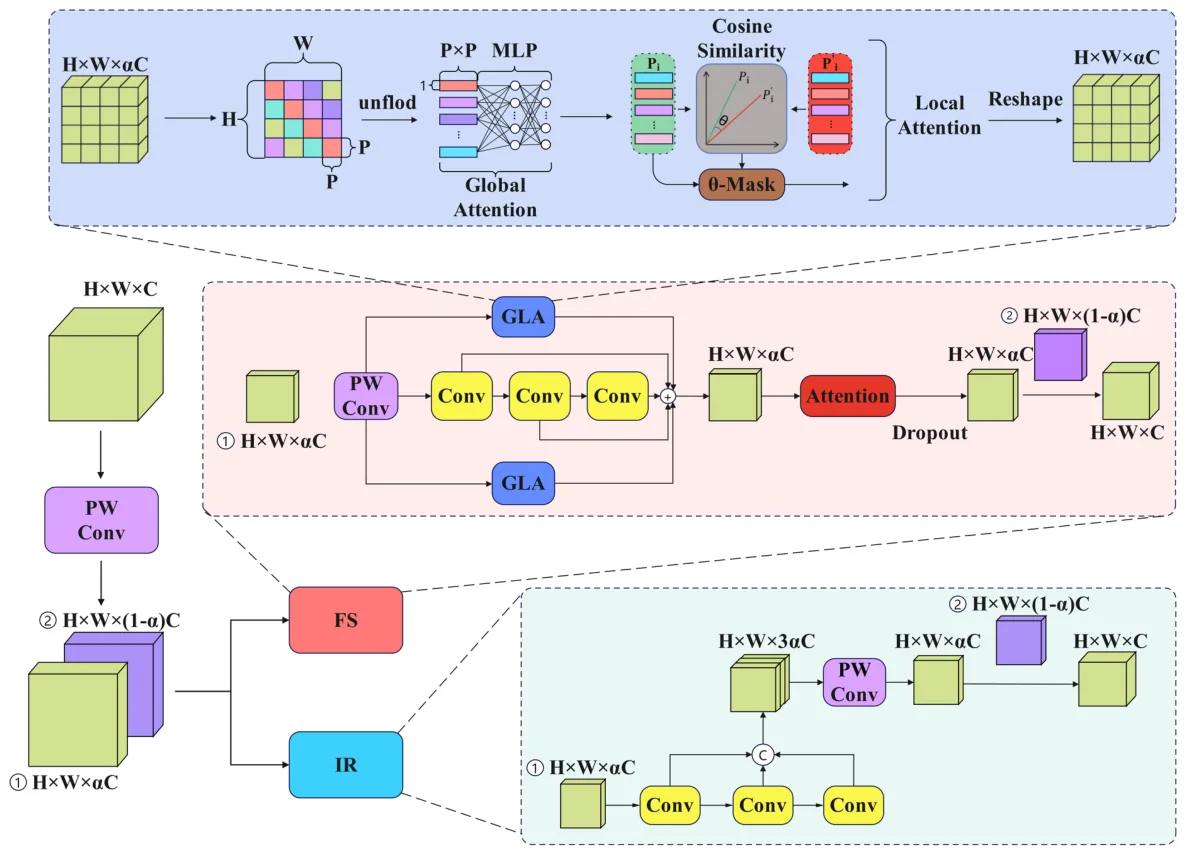
The detailed structure of DRM. This module includes two working modes: information retention mode and feature screening mode. The common part of both modes is that three convolution modules are used to extract features, while the feature screening mode enables multiple attention mechanism layers.

The detailed architecture of the DRM is illustrated in Figure 4. It follows a split-transform-merge strategy. The input feature map, $F\in R^{H\times W\times C}$, is first projected via a 1×1 convolution and then split along the channel dimension into two parts: $F_{1}\in R^{H\times W\times \alpha C}$ and $F_{2}\in R^{H\times W\times (1-\alpha )C}$. The hyperparameter α determines the channel ratio between $F_{1}$ and $F_{2}$. $F_{1}$ is processed further, while $F_{2}$ remains unchanged. This design aims to preserve the original details in certain channels for subsequent network stages. Crucially, the processed ($F_{1}$) and unprocessed ($F_{2}$) channels are fused at the end of the module. This mechanism ensures that fine details are never completely destroyed by shallow DRM layers, regardless of the operating mode, thereby preventing small targets from being entirely erased during processing.

In the IR mode, the processed portion $F_{1}$ passes through three sequential 3×3 convolutional modules. Each module consists of a convolutional layer, a normalization layer, and an activation layer. Their successive application yields feature maps with receptive fields of 3×3, 5×5, and 7×7. Concatenating these three maps produces a tensor with 3αC channels. A pointwise convolution (PW Conv) module then reduces the channel count back to αC. This result is concatenated with $F_{2}$, and another pointwise convolution fuses the information across the channel dimension. In this state, nearly every value in the input feature map generally influences the output, ensuring that low-level details are not entirely lost.

In the FS mode, $F_{1}$ is processed in parallel by two pathways. The first is a convolutional module comprising three 3×3 kernels. The second is a global–local attention module, which assesses the importance of feature blocks from both global and local perspectives to enhance regions likely to contain objects. Specifically, this module first partitions the feature map into multiple P × P blocks along the spatial dimensions, which are then flattened. A multilayer perceptron (MLP) calculates and assigns an importance weight to each block. While the elements within a block are adjacent by definition, the fully connected layers establish links between non-adjacent blocks, compensating for the limited receptive field of the convolutional operations. The weighted blocks are then evaluated via cosine similarity against a set of trainable prototype blocks to further gauge their relevance based on local characteristics. The final output is reshaped to the original $F_{1}$ dimensions: $F\in R^{H\times W\times C}$. The results from the global–local attention module and the convolutional module are summed, processed by a pointwise convolution, and subsequently passed through spatial and channel attention layers. A Dropout layer is applied to randomly disable nodes, enhancing generalization. Similar to the IR mode, the final processed $F_{1}$ is concatenated with $F_{2}$ and fused via pointwise convolution. The FS mode employs extensive attention mechanisms to compress large amounts of irrelevant information. However, during the initial training phase, information pertaining to the detection targets may also be suppressed. Therefore, the FS and IR modes must be used in tandem. The IR mode supplies essential detail to the network architecture, while the FS mode sharpens its focus on potential detection targets.

### 3.3. Information Retention and Feature Screening Synergistic Network

Detecting aviation ground safety and protective devices presents two major challenges in object detection. First, a single image typically contains multiple tool instances, some of which are notably small and prone to losing fine details during repeated downsampling. Second, the tools exhibit low semantic relevance to their background, where complex background textures can significantly interfere with detection. To address these issues and improve both the precision and recall for aviation ground safety and protective devices, we propose the Information Retention and Feature Screening Synergistic Network (RS-Net). Building upon the theoretical mapping and the DRM design established above, the RS-Net instantiates this dual-branch synergy with clear architectural assignments. The main branch is constructed using the FS-mode DRM and pooling layers to specifically target the minimization of I(X;T), enabling aggressive yet effective compression of background interference. In parallel, the auxiliary branch is constructed using the IR-mode DRM and convolutional layers to specifically target the maximization of I(T;Y), ensuring the preservation of fine-grained spatial gradients. This explicit allocation of theoretical objectives to distinct network components allows RS-Net to approximate the optimal information bottleneck trade-off during collaborative training. Although our RS-Net inherits the dual-branch training paradigm from YOLOv9, the design philosophy and intended purpose of the neural network architecture are fundamentally different. In YOLOv9, the auxiliary branch serves primarily as a gradient propagation pathway under the programmable gradient information mechanism, focusing on information retention without explicitly considering the compression-preservation trade-off. In our RS-Net, by contrast, the auxiliary branch equipped with IR mode actively preserves fine-grained spatial details (maximizing I(T;Y)), while the main branch equipped with FS mode employs multiple attention mechanisms to enhance target features (minimizing I(X;T)). This decoupling of the two conflicting objectives, grounded in information bottleneck theory, is the fundamental distinction from YOLOv9’s fixed auxiliary design. As shown in Figure 2, RS-Net consists of three key components: a backbone network, a main detection branch, and an auxiliary detection branch. The architectural details are elaborated below.

The backbone network is constructed by sequentially arranging the FS-mode DRM and max-pooling layers. Taking an input $X_{\mathrm{input}}\in R^{B\times H\times W\times 3}$, its final layer produces an output $X_{\mathrm{output}}\in R^{B\times H/16\times W/16\times C}$, achieving a total downsampling factor of 16 through four stages of DRM and pooling operations. Both pooling and convolution are common downsampling techniques. While convolutional layers are trainable, pooling layers offer greater efficiency and robustness. Max-pooling, in particular, provides strong translation invariance, which helps reduce the model’s sensitivity to background variations. Although max-pooling aggressively discards spatial details, the backbone network leverages this property to effectively compress irrelevant feature information, extracting discriminative features of the target objects with relatively low computational cost.

The auxiliary branch is composed of IR-mode DRM, convolutional layers, Composite Backbone Linear (CBLinear), and Composite Backbone Fusion (CBFuse) modules. It shares the same input with the backbone network but employs convolutional operations for downsampling. Convolutions are, under certain conditions, invertible and can adaptively learn task-specific weights, but they come with higher computational costs and a greater risk of overfitting compared to pooling. The main function of this branch is to preserve original, high-resolution information, ensuring that fine details are not lost to aggressive downsampling or attention-based filtering. The CBLinear module extracts multi-scale feature maps from three different levels of the backbone and projects them into corresponding dimensions. The CBFuse module then fuses these projected features with the feature maps from the auxiliary branch at the same scales. Together, CBLinear and CBFuse facilitate bidirectional interaction across feature scales and bridge the auxiliary branch with both the backbone and the main detection branch, effectively enabling implicit knowledge distillation from the auxiliary branch to the main branch during training.

The main detection branch adopts a Path Aggregation Network (PAN) architecture, integrating FS-mode DRM, convolutional layers, deconvolutional layers, and MDCR (Multi-Dilated Channel Refiner) module. PAN introduces an additional bottom-up pathway that directly and rapidly transmits fine-grained pixel-level information (e.g., edges and textures) from lower-level feature maps to higher-level ones. This design significantly alleviates the problem of small-object information loss in deep networks. Simultaneously, its adaptive feature pooling mechanism allows each candidate object to aggregate the most relevant contextual information from feature maps across all scales. This bidirectional, multi-scale information flow markedly enhances the model’s localization accuracy and recognition robustness for small targets. Compared with FPN or BiFPN, PAN is selected here because its additional bottom-up pathway provides a more direct route for low-level spatial details to reach high-level layers, mitigating the inevitable information loss during deep feature extraction that is critical for precise localization. The MDCR module further augments the branch’s capability by using multi-head dilated depthwise convolutions: it divides the feature channels into four groups, applies different dilation rates to capture multi-scale spatial patterns within each group, and then cross-recombines and fuses the groups via lightweight pointwise convolutions. This process enhances the distinctiveness between small targets and the background, improving detection accuracy without increasing the parameter count. Since the auxiliary branch is only active during training, the main branch must inherently balance the dual capabilities of compressing irrelevant information and preserving crucial target features, resulting in its strong overall performance.

### 3.4. Data Collection and Annotation

To construct a professional and practically meaningful benchmark dataset, we collaborated with an airline company and collected a large set of real-world images captured during their daily ground inspection and maintenance operations. These images are part of the tool inventory procedure: after each maintenance or inspection task, ground staff place the retrieved equipment on the aircraft carpet or hangar floor and take photographs for archival and verification purposes. This practice serves as a critical safeguard against tools accidentally left behind, ensuring that all equipment is accounted for before takeoff. Importantly, all collected images are completely raw, no cropping, filtering or any other preprocessing has been applied, so that the data faithfully preserve the original working conditions, including natural variations in scale, lighting, and occlusion. This design choice, while inevitably limiting the total data volume, guarantees that our dataset reflects genuine operational challenges rather than idealized lab scenarios.

**Figure 5:**
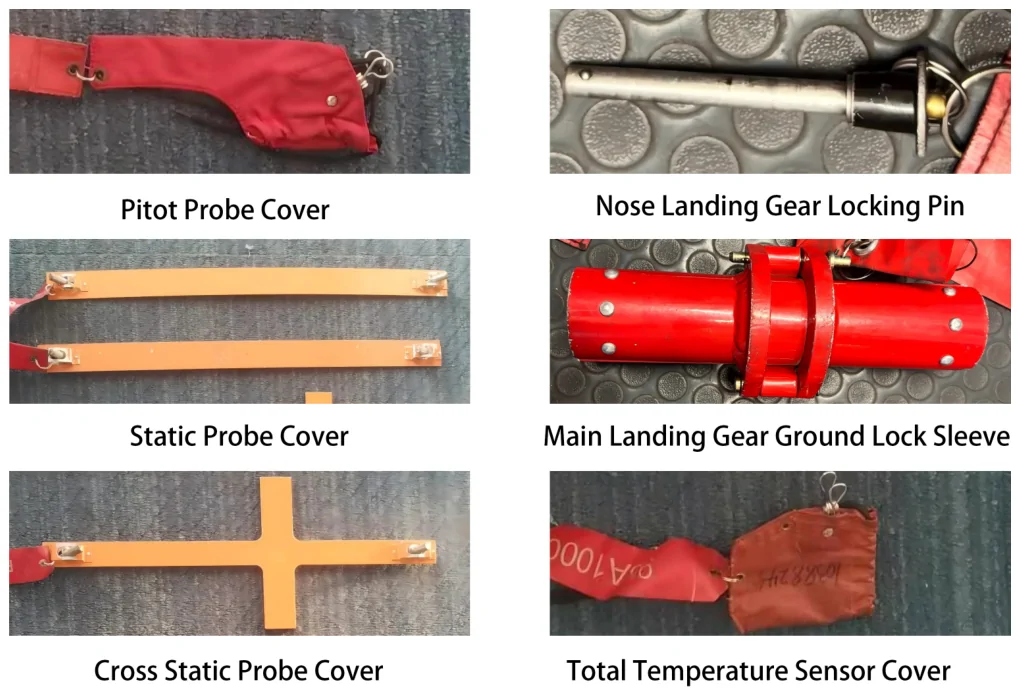
Images of aviation ground safety and protective devices in the dataset. They include six types of tools used in the daily maintenance of a certain airline, namely cross static probe cover, static probe cover, pitot probe cover, total temperature sensor cover, main landing gear ground lock sleeve, and nose landing gear locking pin.

We then manually annotated all images using the LabelImg plugin, following a rigorous protocol. As shown in Figure 5, the annotation covers six categories of aviation ground safety and protective devices: Cross Static Pressure Probe Cover, Static Pressure Probe Cover, Pitot Probe Cover, Total Temperature Sensor Cover, Main Landing Gear Ground Lock Sleeve, and Nose Landing Gear Locking Pin. For each instance, we drew the tightest axis-aligned bounding box that encloses the smallest rectangle surrounding the target; for partially occluded objects, only the visible parts were labeled. Every target in every image was exhaustively marked without omission. To ensure high labeling quality, all annotations were double-checked by two independent annotators, and only boxes with an Intersection over Union (IoU) exceeding 95% were accepted; in cases of disagreement below this threshold, final decisions were made in consultation with airline engineers. The annotated files were saved in YOLO TXT format, containing class IDs and normalized bounding box coordinates. To facilitate compatibility with various detection frameworks, we additionally developed a conversion script that transforms the YOLO-format annotations into both VOC and COCO formats, enabling fair comparisons with other models.

**Figure 6:**
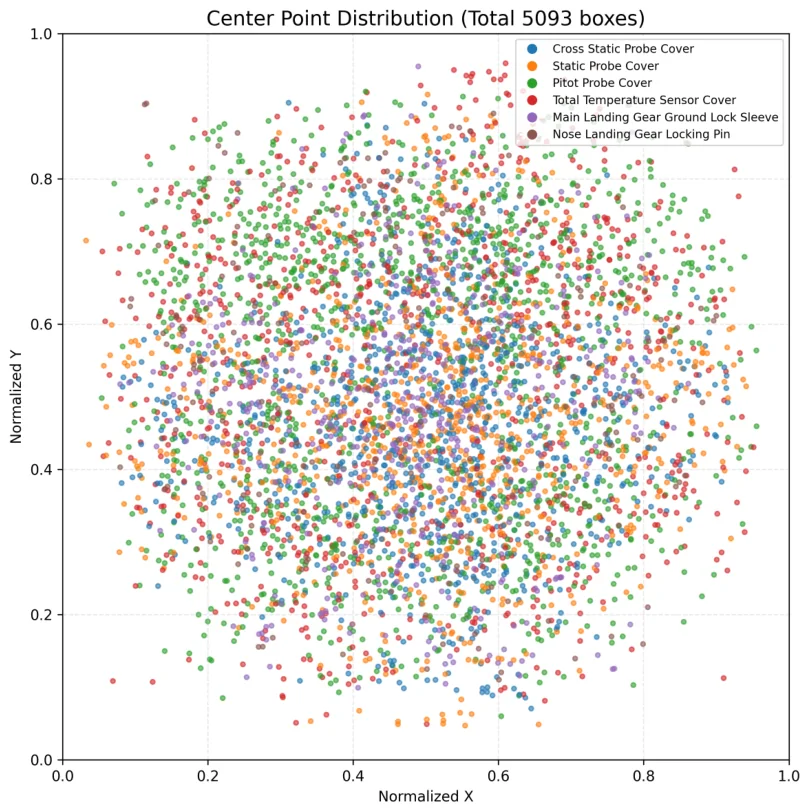
Scatter plot of bounding box center distribution in the dataset. Each dot represents the normalized center coordinate (x,y) of an annotated bounding box, where both axes range from 0 to 1 corresponding to image width and height. Different colors indicate different classes. The semi-transparent overlay reveals density concentrations.

The resulting dataset comprises 766 labeled images with a total of 5093 instances. The average image width is 1458 pixels and average height 1819 pixels. The distribution of these six categories is as shown in the Table 1. Among them, Cross Static Pressure Probe Cover has 969 instances, Static Pressure Probe has 960 instances, Pitot Probe Cover has 1389 instances, Total Temperature Sensor Cover has 952 instances, Main Landing Gear Ground Lock Sleeve has 568 instances, and Nose Landing Gear Locking Pin has 255 instances. It is worth noting that, in line with the airline’s current operational protocol, most objects are placed vertically and with minimal overlap. This is because the existing deployed detector lacks robustness against severe occlusion and arbitrary orientations, so ground personnel are instructed to separate and align equipment before photography to ensure reliable identification. Nevertheless, the dataset still retains considerable diversity, as a substantial portion of images contain objects placed at angles, upside down, or with slight overlaps due to human operational variability. This inherent diversity makes the dataset a true reflection of actual working scenarios and provides a meaningful testbed for evaluating model robustness. The spatial distribution of bounding box centers across the image plane is shown in Figure 6, with different colors representing different classes.

## 4. Experiments

### 4.1. Training Setting

We conducted a comprehensive evaluation of the RS-Net on our self-built dataset of aviation ground safety and protection equipment. The GPU used was Tesla V100-PCIE-32 GB with NVIDIA driver version 560.35.03 and CUDA version 12.6. All experiments were conducted under the same hardware environment and software framework. For model training, we employed the Stochastic Gradient Descent (SGD) optimizer with a batch size of 16 for a total of 300 epochs. The initial learning rate was set to 0.01, and we adopted the OneCycleLR scheduler with the final learning rate also fixed at 0.01. The momentum coefficient was 0.937, and we used a warmup period of 5 epochs. The input images were resized to 640 pixels, and we set a patience of 100 epochs for early stopping. Additionally, the automatic mixed precision (AMP) gradient scaler was configured with a scale factor of 2048.

This study evaluates the model’s performance on aviation ground safety and protective devices using precision, recall, $mAP_{0.5}$, and $mAP_{0.5:0.95}$. Precision, which reflects the model’s capability to correctly identify target instances, is defined as

(4) $$\mathrm{Precision}=\frac{TP}{TP+FP},$$

where TP and FP denote the numbers of true positives and false positives, respectively. Recall, which indicates whether the model misses any detections during inspection, is expressed as

(5) $$\mathrm{Recall}=\frac{TP}{TP+FN},$$

where FN represents the number of false negatives. Since precision and recall are often inversely correlated, mAP is employed to measure their combined performance. Specifically, $mAP_{0.5}$, as shown in Equation (6), is the mean average precision across multiple categories at a single fixed IoU threshold (τ=0.5):

(6) $${\mathrm{mAP}}_{0.5}=\frac{1}{N}\sum_{i=1}^{N}AP_{i}^{0.5},$$

where *N* denotes the number of classes. $mAP_{0.5:0.95}$, given by Equation (7), is the average mAP computed over a set of continuously varying IoU thresholds (τ ranging from 0.50 to 0.95 with a step size of 0.05):

(7) $${\mathrm{mAP}}_{0.5:0.95}=\frac{1}{NT}\sum_{i=1}^{N}\sum_{t\in \{0.5,0.55,\ldots ,0.95\}}AP_{i}^{t}.$$

Finally, the F1 score, a statistical metric used to evaluate the combined performance of precision and recall from a harmonic perspective, is formulated as

(8) $$F1=2\cdot \frac{Precision\cdot Recall}{Precision+Recall}.$$

In these equations, TP, FP, and FN represent the quantities of true positives, false positives, and false negatives, respectively; *N* denotes the number of classes; and $AP_{i}^{t}$ indicates the average precision of the *i*-th class at an IoU threshold of *t*.

### 4.2. Comparison Experiments

**Figure 7:**
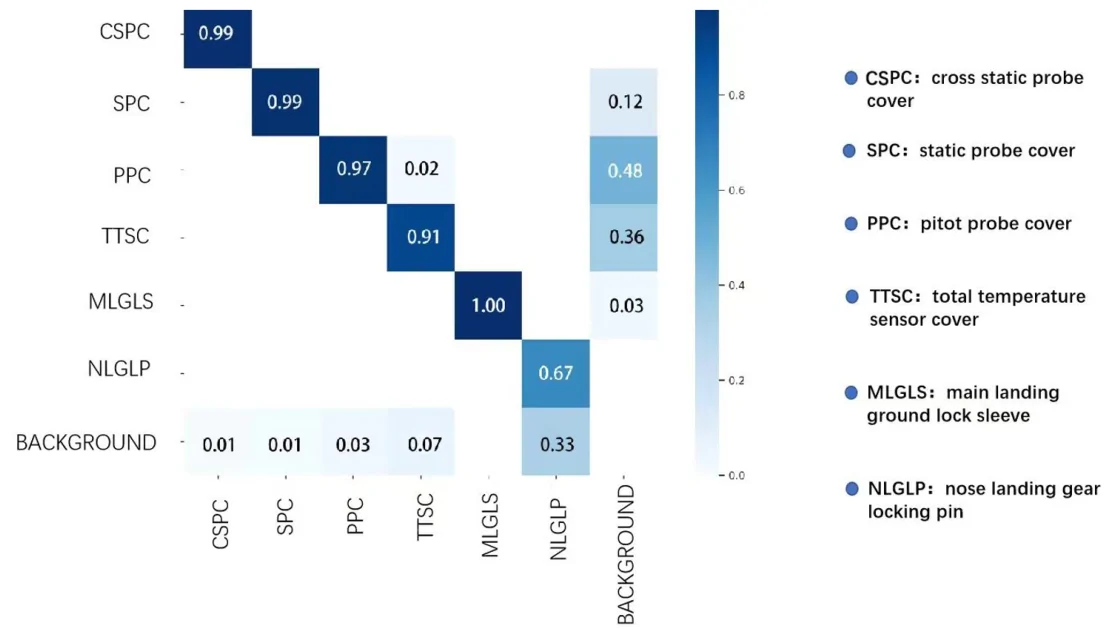
Confusion matrix. This graph shows the detection results of RS-Net for various aviation ground safety and protective devices.

To comprehensively evaluate the effectiveness of the proposed RS-Net model, we conducted extensive comparative experiments against the current mainstream object detection models on our self-built aviation ground safety and protective devices dataset. The models selected for comparison encompass representative one-stage and two-stage architectures, including multiple variants of the YOLO series, two RT-DETR series models, and Faster R-CNN. The experiments employed a unified training–validation split, training hyperparameters, and hardware environment to ensure a fair comparison. The evaluation metrics included Precision, Recall, F1-score, mean Average Precision under different IoU thresholds ($mAP_{0.5}$, $mAP_{0.5:0.95}$), as well as the number of Parameters and computational complexity, aiming to holistically assess the balance between model performance and practical utility. The experimental results are presented in Table 2, and Figure 7 is the confusion matrix of the results.

First, regarding detection accuracy, the RS-Net model proposed in this paper demonstrates a comprehensive and significant lead. It achieves a precision of 0.956, a recall of 0.937, and an F1-score of 0.946, which are superior to all comparative models. This result indicates that RS-Net effectively reduces both false positives and missed detections. In terms of the comprehensive performance metric ($mAP_{0.5}$), RS-Net leads with a score of 95.6%. Furthermore, on the more challenging $mAP_{0.5:0.95}$ metric, which imposes more stringent localization requirements, RS-Net also ranks first with a score of 63.4%. This fully demonstrates that RS-Net not only performs excellently under lenient evaluation criteria but also exhibits greater robustness and precision in real-world scenarios demanding highly accurate bounding boxes.

Second, concerning model efficiency, RS-Net maintains a reasonable model complexity while achieving a breakthrough in detection performance. Although its computational cost is higher than that of single-branch networks, it is lower than YOLOv9, which also employs dual-branch training. Its parameter count is also lower than that of all compared models. This suggests that the performance gain of RS-Net does not stem merely from an increase in parameters but rather benefits from the higher information utilization efficiency enabled by its novel architecture. It is particularly noteworthy that the ’main-auxiliary’ dual-branch design of RS-Net introduces additional computation only during the training phase. During inference, only the main branch is used, thereby ensuring detection speed in practical deployment.

The superior performance of RS-Net in aviation ground safety and protective devices detection can be attributed to two main reasons. First, addressing the characteristics of varying sizes and shapes among the tools, the FS-mode DRM employs global–local attention layers to effectively extract features from different tools and distinguish them from background features. This is particularly beneficial for small tools, such as the locking pin, where the DRM can extract information from limited feature vectors, overcoming the limitations of insufficient receptive field stacking in traditional convolutions for extracting features of small objects. Second, to tackle the problem of sparse contextual information in the aviation ground safety and protective devices detection task, the backbone network of the DRM adopts pooling-based downsampling. While this method significantly disrupts information in the feature maps, it enables the rapid and efficient extraction of region features that are comprehensively enhanced by the attention layers within the DRM, thereby quickly compressing most of the useless background information. The information loss incurred by pooling-based downsampling is compensated for by an auxiliary branch that employs convolution-based downsampling to supplement details. Although the auxiliary branch has weaker feature extraction capability, it provides the main branch with more comprehensive features of the detection targets, thereby encouraging the main branch to concentrate on objects that are easily overlooked.

### 4.3. Visualized Analysis

To gain deeper insights into the decision-making mechanism of the proposed RS-Net and to qualitatively verify that the model indeed focuses on the critical regions of aviation ground safety and protective devices, we employed Gradient-weighted Class Activation Mapping (Grad-CAM) for visual interpretation. Grad-CAM generates coarse localization maps by utilizing the gradient information flowing into the final convolutional layer of the network, highlighting the regions that are most influential for the final classification decision.

**Figure 8:**
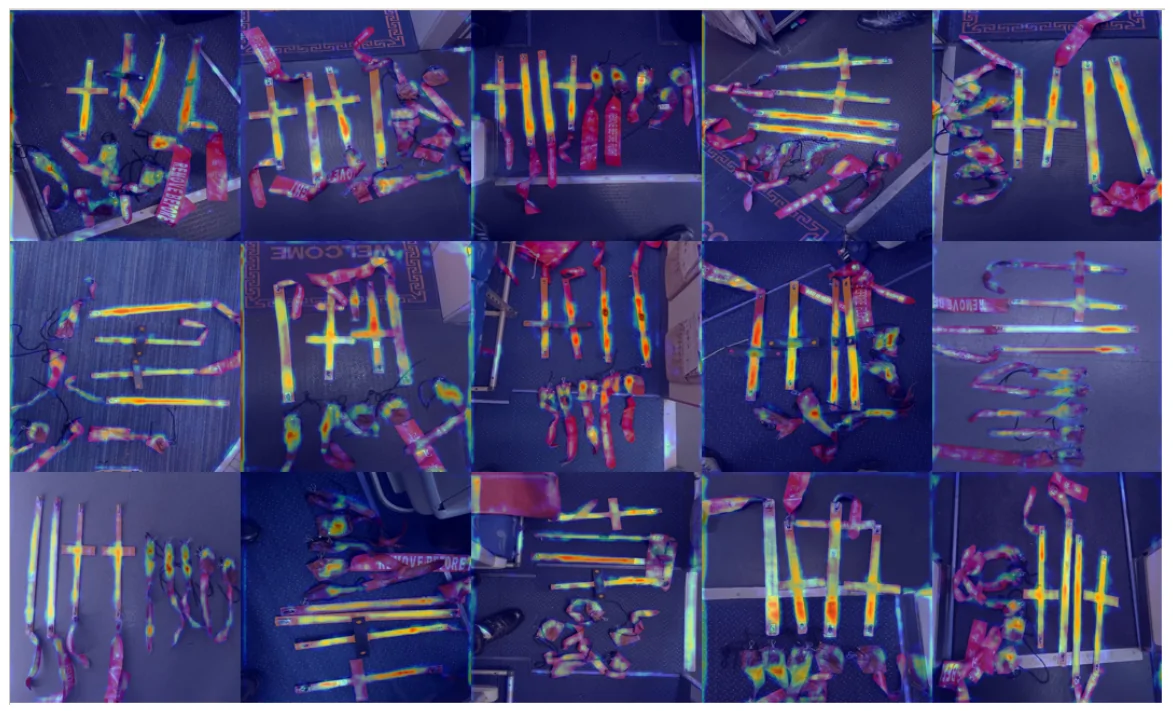
Grad-CAM heatmaps. The warmer colors indicate high-response regions. The heatmap activations cover the majority of the key target areas, demonstrating that the model possesses reliable spatial localization capability and its decision basis is broadly consistent with the expected features.

Figure 8 presents representative Grad-CAM heatmaps overlaid on the original images from our self-constructed dataset. As shown in Figure 8, the model’s activated regions predominantly concentrate on the central body of the tools, rather than being distracted by the carpet texture or the surrounding background. This indicates that the Feature Screening (FS) mode within the main branch effectively suppresses irrelevant background noise, enabling the network to focus on the discriminative morphological characteristics of the target objects. Furthermore, despite significant variations in object scale and placement angles across different samples, the activated heatmaps consistently cover the complete physical extent of the devices, demonstrating the model’s robust localization capability. This behavior aligns with the theoretical objective of minimizing I(X; T), as the network successfully compresses redundant environmental information while retaining features essential for accurate detection.

**Figure 9:**
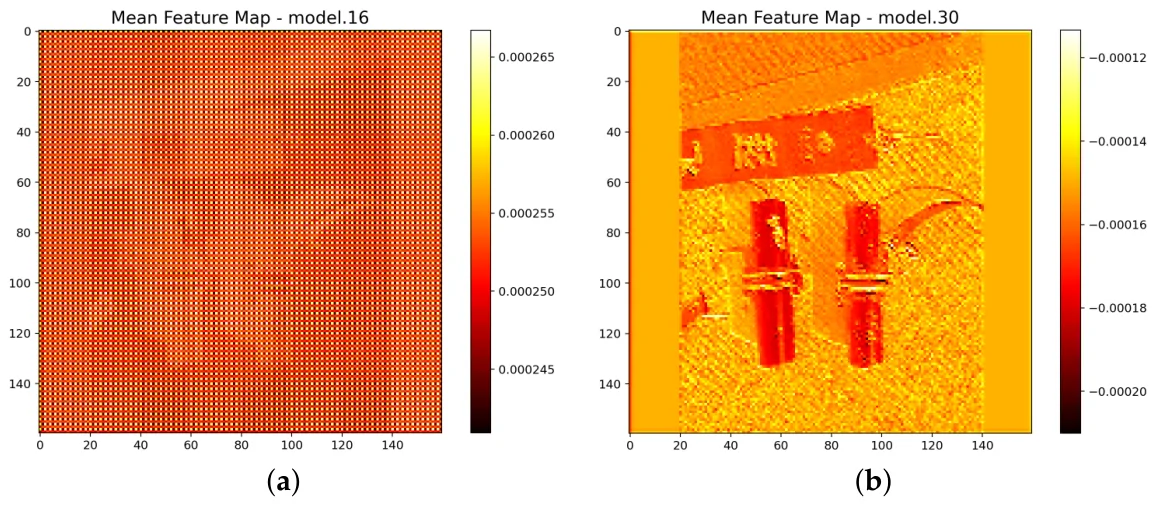
Key visualized feature maps of RS-Net. (**a**) Mean feature map of the main branch. (**b**) Mean feature map of the auxiliary branch. The main branch extracts high-level semantic information that provides the basis for category judgment, while the auxiliary branch extracts low-level semantic information such as edges, colors, and textures.

In addition to the qualitative localization evidence provided by Grad-CAM, we further conducted a quantitative analysis of information flow by examining the intermediate feature maps of the dual branches. As shown in Figure 9, the averages of the shallow feature maps across all channels from the main branch and the auxiliary branch are displayed, which correspond to the outputs from the 16th and 30th layers of RS-Net, respectively. The information entropy [48] of a discrete random variable *X* is defined by

(9) $$H\left(X\right)=-\sum_{x\in X}p\left(x\right)\log_{2}p\left(x\right),$$

where X is the set of possible values for *X*, and p(x) is the probability mass function. For an image *I*, the entropy can be calculated as

(10) $$H\left(I\right)=-\sum_{i=0}^{255}p_{I}\left(i\right)\log_{2}p_{I}\left(i\right),$$

where $p_{I}\left(i\right)$ denotes the probability of intensity value *i* occurring in image *I*, estimated empirically from the normalized pixel intensity histogram. Similarly, the mutual information between two random variables *X* and *Y* is formulated as

(11) $$I\left(X;Y\right)=\sum_{x\in X}\sum_{y\in Y}p\left(x,y\right)\log_{2}\left(\frac{p(x,y)}{p(x)p(y)}\right),$$

and the mutual information between two images $I_{1}$ and $I_{2}$ is given by

(12) $$I\left(I_{1};I_{2}\right)=\sum_{i=0}^{255}\sum_{j=0}^{255}p_{I_{1},I_{2}}\left(i,j\right)\log_{2}\left(\frac{p_{I_{1},I_{2}}\left(i,j\right)}{p_{I_{1}}\left(i\right)p_{I_{2}}\left(j\right)}\right).$$

Based on these formulas, the information entropy of the original image is approximately 7.672 bits, whereas the average feature maps of the main branch and auxiliary branch are about 7.688 bits and 6.411 bits, respectively. The mutual information between the average feature map of the main branch and the original image is approximately 0.047 bits, while that between the average feature map of the auxiliary branch and the original image is about 0.189 bits. These entropy and mutual information estimates are obtained using non-parametric empirical distributions derived from normalized pixel-intensity histograms, which avoids imposing any parametric assumptions on the feature representations. The substantially higher mutual information retained by the auxiliary branch numerically corroborates its role in preserving fine-grained spatial details throughout the training process. In contrast, the lower mutual information of the main branch indicates that it prioritizes the abstraction of high-level semantic features over the retention of low-level pixel details. This quantitative comparison provides direct evidence that the auxiliary branch effectively fulfills its designated information retention function, complementing the main branch’s feature screening objective within the information bottleneck framework.

**Figure 10:**
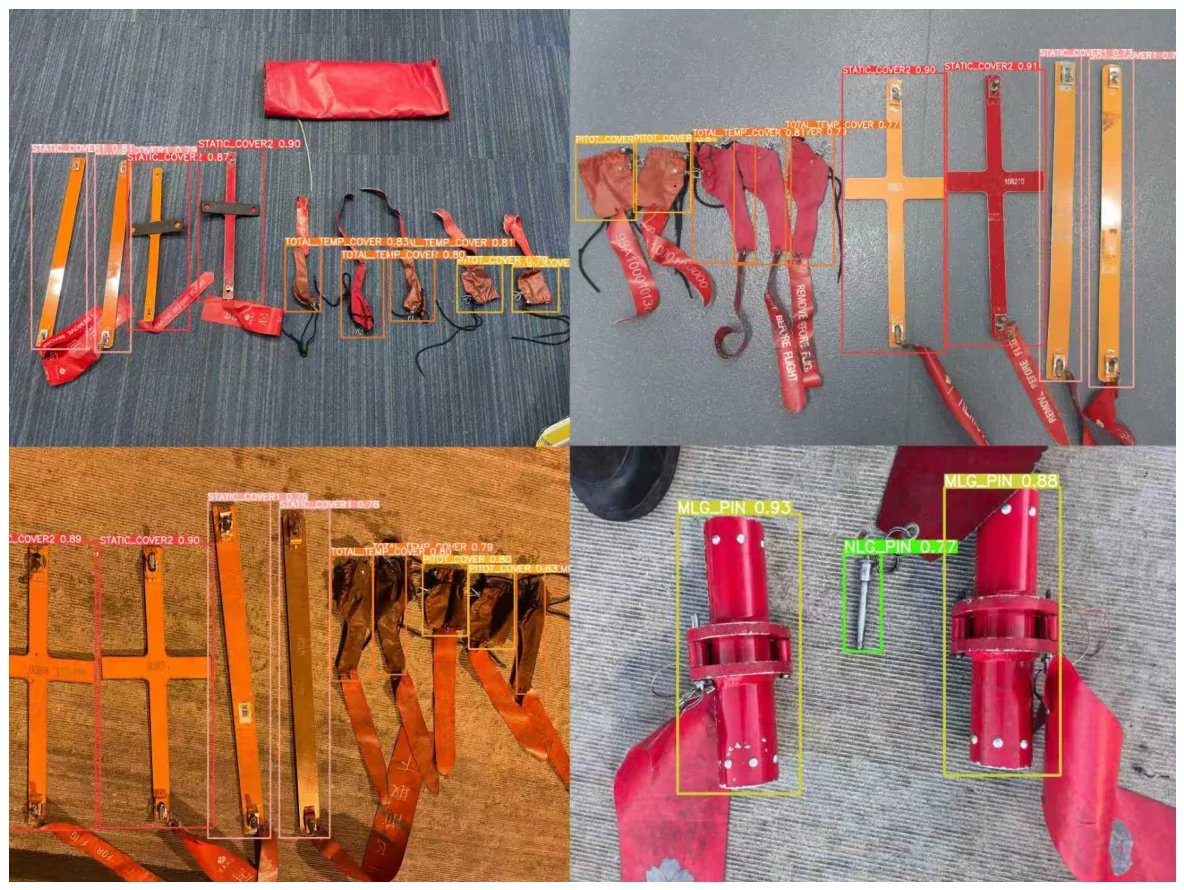
Experimental results. Visualization of the final detection results of RS-Net.

To provide intuitive qualitative evidence for the quantitative results, Figure 10 visualizes representative detection outcomes of RS-Net on the aviation ground safety and protective devices dataset. It can be observed that RS-Net reliably localizes and classifies all six categories of devices under various conditions. These qualitative visualizations corroborate the quantitative metrics reported in Table 2, confirming that RS-Net achieves both high precision and robust recall in practical deployment scenarios.

### 4.4. Generalization Experiment

To comprehensively evaluate the generalization capability and domain adaptability of the proposed method, we conducted generalization performance experiments on a public aircraft skin damage dataset. This dataset focuses on the detection of aircraft surface defects such as corrosion and scratches. Its target characteristics share similarities with aviation ground safety and protective devices, such as small object size and complex backgrounds. However, it exhibits significant differences in damage morphology, texture appearance, and contextual environment. Therefore, it serves as an effective testbed for examining the model’s transfer and generalization potential in unseen aviation-specific scenarios. The experiments maintained training strategies and parameter settings consistent with the main experiments. All comparative models were retrained and evaluated under identical conditions to ensure a fair comparison.

Table 3 presents the performance comparison results of various models on this dataset. The overall trend indicates that the proposed RS-Net still maintains a leading position across multiple metrics. Specifically, RS-Net achieved the highest scores on comprehensive metrics, with an F1-score of 0.746, $mAP_{0.5}$ of 0.787, and $mAP_{0.5:0.95}$ of 0.471. This demonstrates its strong overall capability even in cross-task scenarios. Regarding the Precision metric, RS-Net retains an advantage over other models, indicating that its architecture effectively suppresses false positives while maintaining high localization accuracy. In terms of Recall, RS-Net’s advantage over other models is less pronounced, yet it remains highly comparable to models achieving high recall rates. RS-Net significantly improves Precision and mean Average Precision while maintaining a relatively high Recall, suggesting that its feature selection mechanism can better adapt to background interference and target shape variations common across different aviation vision tasks.

The generalization experiment results indicate that RS-Net not only excels in its originally designed task of aviation ground safety and protective devices detection but also maintains strong competitiveness in the related yet distinct task of aircraft skin damage detection. This validates the effectiveness of the proposed dual-branch collaborative framework and the DRM in capturing generalizable visual features. The performance advantage stems from the information preservation branch’s consistent maintenance of spatial details and the feature selection branch’s reinforcement of discriminative features. Their synergy enables the model to better adapt to challenges commonly encountered in the aviation domain, such as small targets, low contrast, and complex backgrounds, demonstrating strong cross-task transfer potential.

### 4.5. Ablation Studies

To verify the synergistic effect of the proposed Dual-State Region Refinement Module and the auxiliary branch within the RS-Net framework, we further designed ablation experiments targeting the DRM and the auxiliary branch. These experiments aim to systematically evaluate the individual contributions of the DRM and the auxiliary branch to model performance and to investigate the synergistic enhancement achieved by their combined use. The overall network architecture and other hyperparameters were kept constant, with adjustments made only to whether the DRM was enabled (if not, the baseline YOLOv9’s feature extraction module RepNCSPELAN4 was used) and whether the auxiliary branch was retained. The experimental results are shown in Table 4.

The results indicate that both the DRM and the auxiliary branch play important roles in improving model detection performance, and their combination yields a significant synergistic effect. When the model contains neither the DRM nor the auxiliary branch, all metrics are at their lowest levels, particularly showing weakness in Precision and $mAP_{0.5}$. From the perspective of information bottleneck theory, the absence of a dedicated auxiliary branch causes progressive loss of fine-grained details during downsampling, thereby failing to maximize the mutual information between the learned representation *T* and the target labels *Y*. Moreover, the baseline module alone cannot effectively balance the trade-off between compressing task-irrelevant information (minimizing I(X;T)) and preserving target-related information (maximizing I(T;Y)). Consequently, the resulting representation *T* is suboptimal, suffering from both excessive background interference and insufficient target details.

Introducing only the auxiliary branch without the DRM leads to a significant improvement in Precision to 0.942, but Recall drops to 0.867. The auxiliary branch, which employs convolutional downsampling rather than pooling, maintains a higher mutual information with the original image. This retention of fine-grained details enhances the model’s ability to capture target cues, thereby increasing precision. However, without a dedicated feature screening mechanism, the network struggles to suppress irrelevant background features, leading to a higher false-negative rate and lower recall. In information bottleneck terms, this configuration increases I(T;Y) to preserve more target information, but fails to sufficiently reduce I(X;T) to remains redundant background information, resulting in an imbalanced optimization.

Using only the DRM without the auxiliary branch results in significant improvements across Precision, Recall, and all mAP metrics. The DRM exhibits stronger information extraction capability than the baseline module, effectively enhancing the model’s ability to capture discriminative features and suppress background noise. However, despite this improvement, some fine-grained spatial details—especially for small objects—are still inevitably lost during deep abstraction due to the lack of a separate pathway dedicated to detail preservation. This limits further performance gains, particularly in Recall.

When both the DRM and the auxiliary branch are enabled, the model achieves optimal overall performance: Recall increases to 0.937, the F1-score reaches 0.946, and $mAP_{0.5}$ and $mAP_{0.5:0.95}$ rise to 0.955 and 0.635, respectively. This configuration realizes a true synergistic effect under the information bottleneck framework. The auxiliary branch acts as a dedicated information retention pathway, providing high-mutual-information gradients during training to increase I(T;Y), ensuring that small-object details are not erased. Meanwhile, the main branch, equipped with DRM, aggressively compresses background redundancy and focuses on discriminative features. The implicit knowledge distillation from the auxiliary to the main branch guides the main branch toward a representation that simultaneously maintains sufficient task-relevant information and suppresses irrelevant noise—an optimal point in the IB trade-off. This collaborative design fundamentally addresses the core challenges of aviation ground safety and protective devices detection, such as missed detection of small objects and strong background interference, by decoupling the conflicting objectives of information preservation and compression into two specialized branches. The ablation results thus confirm, at the component level, that the proposed synergistic design is the key architectural factor enabling RS-Net’s superior detection performance.

To further optimize the internal structure of DRM and investigate the impact of the feature split ratio on model performance, we conducted an ablation study on the separation coefficient α. The DRM employs a split-transform-merge strategy, dividing the input feature map along the channel dimension into two parts with channel ratios α and 1−α. The portion with ratio α ($F_{1}$) undergoes processing including multi-scale convolution and attention mechanisms to enhance discriminability, while the remaining portion with ratio 1−α ($F_{2}$) maintains an identity mapping to preserve the original information flow. This experiment aims to determine the optimal capacity allocation between the information processing path, which compresses I(X;T), and the information retention path, which preserves I(T;Y).The results are presented in Table 5.

The experimental results show that the separation coefficient α has a significant and regular impact on model performance. The model achieves the best overall performance when α=0.5, meaning equal channel numbers are allocated to processing and retention. From an information bottleneck perspective, smaller α values allocate excessive channels to identity mapping, resulting in over-retention of redundant background information. This increases I(X;T) and introduces noise, thereby impairing detection precision. Conversely, larger α values devote excessive capacity to attention-driven compression, which aggressively suppresses irrelevant information and consequently decreases I(X;T). However, this inevitably sacrifices fine-grained target details, leading to a reduction in I(T;Y) and causing missed detections of small objects. The balanced configuration α=0.5 achieves an optimal compromise between feature screening and information retention, allowing the network to fully extract and reinforce key features while preserving sufficient spatial detail. This balance yields both high Precision and high Recall, confirming that the equal split provides the most favorable operating point in the IB trade-off for this task.

To validate the effectiveness of different working mode configurations within the proposed dual-branch collaborative design, we conducted an experiment on module modes. This experiment aims to investigate the impact of different combinations of the Information Retention (IR) and Feature Screening (FS) modes in the Main Branch and the Auxiliary Branch on the final detection performance, thereby revealing the roles of each component and the optimal collaboration method within the dual-branch synergy mechanism. The overall network architecture and other hyperparameters were kept consistent, with changes made only to the working modes of the DRM modules in the two branches. The results are shown in Table 6.

The experimental data clearly show performance differences under various configurations. When both the Main and Auxiliary Branches use the Information Retention mode (IR-IR), the model achieves an mAP of only 0.930 and 0.610 for $mAP_{0.5}$ and $mAP_{0.5:0.95}$, respectively. In this configuration, both branches prioritize the preservation of original information over the compression of irrelevant features. From an information bottleneck perspective, this represents a significant imbalance in the optimization objective: the network fails to sufficiently minimize I(X;T), as the dominant identity mappings in both branches leave abundant background noise intact throughout the feature extraction pipeline. Consequently, the representation *T* retains excessive task-irrelevant information, which interferes with the detection head’s ability to discriminate between targets and background, leading to insufficient differentiation of objects and an increased false-positive rate.

Conversely, when both branches use the Feature Screening mode (FS-FS), the model exhibits mediocre performance under stringent IoU thresholds. In this case, both branches aggressively compress the input via attention mechanisms, effectively lowering I(X;T) and achieving high background suppression. However, the absence of any dedicated information retention pathway means that fine-grained spatial details, particularly those of small objects, are also subjected to this compression process. As quantified earlier, the mutual information between feature maps and the original image drops significantly with excessive FS operations. This results in the erosion of target boundaries and the blurring of localization cues, which severely degrades bounding box regression accuracy under high IoU thresholds. The FS-FS configuration thus over-prioritizes compression at the expense of preservation, pushing the network to the opposite extreme of the IB trade-off.

In contrast, when heterogeneous configurations are employed, model performance improves substantially. The combination of Feature Screening for the Main Branch and Information Retention for the Auxiliary Branch (FS-IR) achieves the optimal overall performance: $mAP_{0.5}=0.955$ and $mAP_{0.5:0.95}=0.635$. This configuration aligns precisely with the information bottleneck-guided design philosophy. The FS-mode Main Branch specializes in minimizing I(X;T), actively compressing background redundancy and extracting high-level discriminative features essential for accurate classification and localization. Simultaneously, the IR-mode Auxiliary Branch specializes in preserving I(T;Y), maintaining a high-mutual-information gradient pathway that feeds fine-grained spatial details into the training process and prevents the main branch from discarding critical target cues. This division of labor effectively decouples the conflicting IB objectives into two specialized streams, allowing the network to jointly approach the optimal representation where I(X;T) is sufficiently low and I(T;Y) remains sufficiently high.

The inverse combination, Information Retention for the Main Branch and Feature Screening for the Auxiliary Branch (IR-FS), also performs reasonably well, yielding an $mAP_{0.5}$ of 0.930 and an $mAP_{0.5:0.95}$ of 0.619. However, its performance falls short of FS-IR. In this configuration, the Main Branch retains excessive detail, meaning the final detection head operates on a representation with suboptimal abstraction—too much low-level information introduces noise and increases false positives. Meanwhile, the Auxiliary Branch actively compresses the very details it was originally designed to preserve, rendering its role of limited practical value. Consequently, the main branch receives less effective detail supplementation during training, and the overall representation *T* deviates from the optimal IB point. These results confirm that the FS-IR pairing provides the most favorable division of labor, where the main detection branch focuses on abstraction and discrimination while the auxiliary branch exclusively handles detail preservation, achieving the best possible approximation of the optimal information bottleneck representation.

## 5. Conclusions

This paper proposes RS-Net, a dual-branch collaborative detection network grounded in information bottleneck theory, to address the challenges of detail loss and sparse semantic information in aviation ground safety and protective devices detection. The architecture comprises a feature-screening main branch and an information-retention auxiliary branch, equipped with a configurable DRM module that preserves fine-grained details while enabling deep feature abstraction. Extensive experiments on a self-constructed dataset demonstrate that RS-Net outperforms mainstream detectors across all metrics, achieving gains of 4.531% in F1-score, 2.533% in mAP_0.5_, and 1.429% in mAP_0.5:0.95_ over the YOLOv9 baseline. Additionally, it achieves a slightly higher inference speed (64.44 FPS) compared to YOLOv9 (63.12 FPS). Cross-dataset experiments and ablation studies further confirm its strong generalization capability and the effectiveness of the dual-branch synergistic design.

Despite these advantages, the current study has limitations. The dataset, though representative of real-world operations, exhibits variability in lighting and occlusion that may compromise robustness in less constrained scenarios. Additionally, training and evaluation on data from a single airline may limit cross-scene generalization. Future work will focus on expanding the dataset with greater diversity and exploring domain adaptation strategies to enhance transferability to broader operational environments.

## Figures and Tables

**Table 1:** Category distribution of the constructed aviation ground safety and protective devices dataset.

Detection Target	Amount	Average Width (in Pixels)	Average Height (in Pixels)	Average Area (in Pixels)
Cross Static Probe Cover	969	483	537	124,810
Static Probe Cover	960	472	566	81,351
Pitot Probe Cover	1389	232	262	31,058
Total Temperature Sensor Cover	952	251	239	58,922
Main Landing Gear Ground Lock Sleeve	568	488	523	171,272
Nose Landing Gear Locking Pin	254	193	194	21,158

**Table 2:** Performance comparison of RS-Net with state-of-the-art object detection models. Bold values indicate the best performance.

Model	Param. (M)	FLOPs (G)	Precision	Recall	F1	${\mathrm{mAP}}_{0.5}$	${\mathrm{mAP}}_{0.5:0.95}$
**RS-Net (ours)**	20.5	198.7	**95.639%**	**93.676%**	**95.460%**	**95.639%**	**63.457%**
YOLOv8	76.7	191.9	91.934%	88.749%	90.313%	93.257%	61.807%
YOLOv9	60.8	266.2	89.605%	90.633%	90.116%	92.927%	62.028%
YOLOv10	32.5	139.7	84.209%	86.355%	85.268%	91.351%	59.989%
YOLOv11	48.3	105.6	90.603%	91.715%	91.156%	94.296%	61.022%
YOLOv12	48.3	99.2	95.222%	89.652%	92.353%	92.728%	59.853%
YOLOv13	24.7	88.1	93.156%	89.496%	91.254%	93.616%	61.542%
YOLO26	27.5	86.1	89.447%	88.529%	88.986%	91.263%	59.846%
RTDETR-resnet101	61.8	191.4	86.958%	73.806%	79.844%	84.342%	53.551%
RTDETR-l	32.0	103.5	86.045%	79.224%	82.494%	84.889%	55.209%
Faster-RCNN	28.3	59.3	82.429%	91.528%	86.741%	82.429%	44.389%

**Table 3:** Performance comparison of RS-Net with state-of-the-art object detection models. Bold values indicate the best performance.

Model	Precision	Recall	F1	${\mathrm{mAP}}_{0.5}$	${\mathrm{mAP}}_{0.5:0.95}$
**RS-Net (ours)**	**80.8%**	69.4%	**74.6%**	78.7%	**47.1%**
YOLOv8	75.0%	68.6%	71.6%	76.4%	44.2%
YOLOv9	75.5%	70.7%	73.0%	**78.9%**	46.4%
YOLOv10	79.5%	69.9%	74.3%	76.0%	45.6%
YOLOv11	77.1%	**70.8%**	73.7%	74.9%	43.2%
YOLOv12	79.8%	69.4%	74.2%	73.5%	42.8%
YOLOv13	79.0%	69.8%	74.1%	77.6%	44.1%
YOLO26	78.4%	69.2%	73.5%	73.9%	42.1%
RTDETR	68.1%	65.5%	66.7%	66.7%	38.5%
Faster R-CNN	51.7%	54.3%	53.0%	63.3%	42.5%

**Table 4:** Ablation studies on the contributions of DRM and auxiliary branch. Bold values indicate the best performance.

DRM	Auxiliary Branch	Precision	Recall	F1	${\mathrm{mAP}}_{0.5}$	${\mathrm{mAP}}_{0.5:0.95}$	Param. (M)	FLOPs (G)
×	×	86.2%	91.8%	88.9%	90.7%	59.6%	9.4	111.7
×	✓	94.2%	86.7%	90.3%	90.8%	59.3%	12.7	143.7
✓	×	**96.2%**	90.3%	93.2%	93.7%	62.3%	14.0	156.8
✓	✓	95.6%	**93.7%**	**94.6%**	**95.5%**	**63.5%**	20.5	198.7

**Table 5:** Impact of channel split ratio α in DRM on detection performance. Bold values indicate the best performance.

α	Precision	Recall	F1	${\mathrm{mAP}}_{0.5}$	${\mathrm{mAP}}_{0.5:0.95}$
0.125	92.2%	91.1%	91.6%	93.7%	62.2%
0.25	96.3%	89.4%	92.7%	95.7%	61.9%
**0.5**	**95.6%**	**93.7%**	**94.6%**	**95.5%**	**63.5%**
0.75	92.7%	90.9%	91.8%	93.4%	60.8%

**Table 6:** Performance comparison of different DRM mode configurations in the dual-branch framework. Bold values indicate the best performance.

Main Branch	Auxiliary Branch	Precision	Recall	F1	${\mathrm{mAP}}_{0.5}$	${\mathrm{mAP}}_{0.5:0.95}$
IR	IR	93.4%	90.2%	91.8%	93.0%	61.0%
FS	FS	91.2%	92.1%	91.7%	93.9%	60.0%
IR	FS	90.6%	89.7%	90.2%	93.0%	61.9%
**FS**	**IR**	**95.6%**	**93.7%**	**94.6%**	**95.5%**	**63.5%**

## Data Availability

The image data used in this study are owned and controlled by Qingdao Airlines Co., Ltd. and are not publicly available due to commercial and privacy restrictions. The data may be accessed upon reasonable request to the corresponding author, subject to the conditions set by the data owner. During the peer-review process, the editorial office and reviewers may internally verify the data solely for the purpose of evaluating the manuscript, but the data shall not be further disclosed, copied, or distributed.

## Abbreviations

The following abbreviations are used in this manuscript:

RS-Net	Information Retention and Feature Screening Synergistic Network
DRM	Dual-State Region Refinement Module
IR	Information Retention
FS	Feature Screening
PAN	Path Aggregation Network
MDCR	Multi-Dilated Channel Refiner
PW Conv	pointwise convolution
CBLinear	Composite Backbone Linear
CBFuse	Composite Backbone Fusion
